# *M.tb-Rv2462c* of *Mycobacterium tuberculosis* Shows Chaperone-like Activity and Plays a Role in Stress Adaptation and Immunomodulation

**DOI:** 10.3390/biology12010069

**Published:** 2022-12-30

**Authors:** Masuma Khawary, Roopshali Rakshit, Aayush Bahl, Pallavi Juneja, Sashi Kant, Saurabh Pandey, Deeksha Tripathi

**Affiliations:** 1 Microbial Pathogenesis and Microbiome Lab, Department of Microbiology, Central University of Rajasthan, Ajmer 305817, India; 2 Department of Biochemistry, School of Chemical and Life Sciences, Jamia Hamdard, New Delhi 110062, India; 3 Department of Immunology and Microbiology, University of Colorado School of Medicine, Anschutz Medical Campus, Aurora, CO 80045, USA

**Keywords:** *Mycobacterium tuberculosis*, trigger factor, PPIase, chaperone

## Abstract

**Simple Summary:**

*Mycobacterium tuberculosis* protein tig (Rv2462c) is present in membrane fraction along with the cytosol. It has been previously reported to assist in the folding of nascent polypeptides. We have investigated the role of *Mycobacterium tuberculosis* tig in stress adaptation; for example, it exhibits roles in thermal and oxidative shock, survival within macrophages, biofilm formation and immunological responses. Our findings establish the contribution of tig to tolerance and adaptation to host-generated stresses, implying its involvement in the infection cycle of *Mycobacterium tuberculosis*. These attributes of Tig highlights its significance in *Mycobacterium tuberculosis* virulence and make it an important target for novel therapeutic interventions against tuberculosis.

**Abstract:**

*Mycobacterium tuberculosis* (*M.tb*)-encoded factors protect it against host-generated stresses and support its survival in the hostile host environment. *M.tb* possesses two peptidyl-prolyl *cis-trans* isomerases and a probable trigger factor encoded by *Rv2462c* which has an FKBP-like PPIase domain. PPIases are known to assist the folding of peptidyl-prolyl bonds and are involved in various cellular processes important for bacterial survival in host-generated stresses. In this study, we aim to functionally characterize *Rv2462c* of *M.tb.* Our data suggest that the trigger factor of *M.tb* exhibits chaperone activity both in vitro and in vivo. Heterologous expression of *M.tb*-*Rv2462c* locus into *Mycobacterium smegmatis* enhanced its survival within macrophages, adaptation to oxidative stress and biofilm formation. *M.tb*-trigger factor has strong immunomodulatory potential and modifies the cytokine profile of the host towards the proinflammatory axis.

## 1. Introduction

Despite our best efforts, *Mycobacterium tuberculosis (M.tb)* is a human peril causing millions of deaths every year due to tuberculosis (TB). Evasion of host defences helps it to survive in hostile macrophages [1]. The World Health Organization (WHO) has estimated that about 25% of the world’s population is affected by latent TB. Though mostly asymptomatic, 5–10% progress into active infections [2]. Efforts to discover new antibiotics against multidrug-resistant *M.tb* has been marked as a global priority by WHO [3]. Various stress responding factors including heat shock proteins, foldases and chaperones contribute to virulence thereby significantly aided in its pathogenicity by immune evasion and colonization of host macrophages [4]. However, the mechanisms employed by the pathogen to survive the stresses of the macrophage microenvironment have not been well elucidated yet. Its better understanding may help in the containment of *M.tb* infection and may suggest novel therapeutic interventions.

Trigger factors across diverse bacteria are essential for the co-translational folding of many nascent polypeptides [5]. The bacterial trigger factor chaperones bind to the 50S ribosomes near the peptide exit channel, enabling its interaction with nascent polypeptides, and facilitating their folding [6,7]. In vitro, the trigger factor refolds denatured RNase T1 and Glyceraldehyde-3-phosphate dehydrogenase (GAPDH) as a prolyl isomerase [8,9].

In an attempt to identify *M.tb* encoded protein responsible for interactions between host and pathogen, stress adaptation, and immunomodulation, we identified and characterized *Rv2462c* locus of *M.tb*. It has an FKBP-like or PPIase domain which may confer chaperone-like activity and is postulated to be involved in cellular processes [10]. This protein has been previously reported to be a probable trigger factor protein of *M.tb* [11]. The percentage amino acid coverage of tig with has 7.1% amino acid sequence identity with PPIase A and 3.8% identity with PPIase B within *M.tb* genome. Prior studies have revealed that *M.tb*-*Rv2462c* is involved in the maintenance of open conformations of newly synthesized proteins, which indicates its chaperonic nature [12]. It has also been observed that nutrient deprivation enhances the expression level of this protein [13]. Inorganic phosphate limitation, especially, is believed to regulate the concentration of *M.tb*-*Rv2462c*. It has been further reported that *M.tb*-*Rv2462c* may contribute significantly to the pathogenicity of *M.tb*, making it significant for further studies [12,14].

In this study, we have characterized the *M.tb*-*Tig* (*Rv2462c*) and explored its immunomodulatory potential and role in promoting *M.tb* survival under intracellular stresses by triggering the host immune system and modifying the cytokine secretion profile of the host, highlighting the significance of the protein in *M.tb* pathogenesis. Here, we have demonstrated that the recombinant *Tig* (*Rv2462c*), when expressed in *E. coli*, displays chaperone-like activity and significantly improves survival chances under high-temperature conditions. When expressed in *M. smegmatis*, it results in enhanced biofilm formation and increased survivability under oxidative stress. Our observations point towards the involvement *of M.tb-Tig* in stress adaptation, which forms an important component of the pathogenicity of *M.tb*.

## 2. Materials and Methods

### 2.1. Bacterial Strains and Cell Lines

*E. coli BL-21* strain from Novagen was used for protein expression. *M.tb-H37Rv* genomic DNA was a gift from Prof. Rakesh Bhatnagar’s laboratory in JNU, New Delhi, India. THP-1 cell was obtained from National Centre for Cell Sciences, Pune, India.

*M. smegmatis* mc^2^ 155 was initially procured from ATCC (Cat#700084). 7H9 broth with 10% OADC and 0.05% tween-80 was used to grow mycobacterium strains.

### 2.2. Cloning and Purification of M.tb-Rv2462c by Ni-NTA Column

*M.tb*-*Rv2462c* gene locus was amplified by using forward (CTAGCTAGCGTGAAGAGCACCGTCGAGCAGTT) and reverse primers (CCGCTCGAGTCGAGTCACGTTGTCGCTTCGTC) and cloned in pET28a vector [15]. From tags of both terminals, only N-terminal poly-his tag was utilized while C-terminal tag was suppressed during expression by stop codon of the cloned gene. The His-tagged *M.tb*-*Rv2462c* gene was expressed in *BL21* cells. Recombinant protein was purified using the Ni-NTA column as described earlier [16]. Then, the eluted fraction was dialyzed to exchange buffer at 4 °C as described earlier [16]. The his-tag has small size and was not cleaved from the rTig after purification.

### 2.3. Enzyme Activity

Enzyme activity of recombinant trigger factor (rTig) was measured using a spectrophotometric assay used previously [15]. In summary, rTig catalyses *cis-trans* isomerization of Ala-Pro bond. The *trans* form is taken as substrate by coupled enzyme α-chymotrypsin to produce chromogenic product. Thus, isomerization of Ala-Pro bond was seen as an enhancement in the absorbance at 390 nm at 15 °C. Absorbance was documented every 1.5 s for 100 s and plotted graphically against time. Control reaction was used in absence of rTig [15].

### 2.4. Chaperone-like Activity of M.tb Rv2462c

Restoration of enzyme activity of heat-denatured proteins is considered a key function of chaperones. To determine if enzyme activity of thermally aggregated NdeI can be recovered by the *M.tb-Rv2462c* protein, 200 ng of the pET22b vector was digested by NdeI with and without thermally aggregated NdeI at 37 °C for 2 h. Heat-denaturation of NdeI was achieved by submerging 10 U of the enzyme at 60 °C for 10 min. The digested plasmid was then electrophoresed in agarose gel containing ethidium bromide stain and visualized by the Gel Doc system (Bio-Rad, Kidlington, UK) [15,17].

To explore the hydrophobicity surface of *M.tb*-rTig, ANS probe was used and monitored by fluorescence spectroscopy [15,18]. Briefly, in presence of rTig, ANS fluorescence was evaluated by excitation at 390 nm and subsequent emission measurements ranging from 450 to 550 nm [17]. The fluorescence of protein-bound ANS dye was measured after mixing 0.5 mg/mL of the recombinant tig with 50 μM ANS and incubated at RT for 30 min. Emission spectrum of the ANS alone was used as control.

Heat-shock of *E. coli* was given as described earlier [19]. Fold survival in the presence and absence of heat shock was calculated, with the vector control (pET22b) being treated as one fold in the normalisation process [17].

### 2.5. Expression of M.tb-Rv2462c in Mycobacterium Smegmatis

To ascertain the impact of *Rv2462c* on the survivability of mycobacterium inside the host, we assayed recombinant *M. smegmatis* strains, expressing *M.tb-Rv2462c*, for growth within THP-1 monocyte cells, in vitro. The *E. coli*-mycobacterium shuttle vector pSTKT having UV15 promoter with tetO sequence (inducible with anhydrotetracycline) was used to construct pST-*Rv2462c*. pST-*Rv2462c* was electroporated into *Mycobacterium smegmatis* mc^2^ 155 as described earlier [20].

### 2.6. Examining In Vitro Growth and Effect of H_2_O_2_

*Mycobacterium smegmatis* strains (Ms_VC, Ms_WT and Ms_tig) were grown in 7H9 broth containing 10% OADC to an OD_600_ of 0.8–1.0. The grown cultures were diluted to a ratio of hundred 1:100. Then, the cultures were grown to an OD_600_ of 0.4 and treated with 7 mM H_2_O_2_ for 3 h. The cultures were diluted 1:100 and plated on 7H10 agar plates.

### 2.7. Intracellular Replication of M. Smegmatis Strains in THP-1 Cells

Human THP-1 monocyte cells cultured in RPMI 1640 medium were differentiated into macrophages by phorbol 12-myristate 13-acetate (PMA) [21] and grown by incubating at 37 °C and 5% CO_2_. *M. smegmatis* cultures (Ms_WT, Ms_tig and Ms_VC) were then used to infect the monolayers of THP-1 for 4 h at a 50 MoI. Then, the infected THP-1 cells were treated with Gentamicin (20 μg/mL) for 30 min. Further, the THP-1 cells were washed twice with RPMI medium and fresh medium was added and incubated for 72 h in CO_2_ incubator. The cells were then taken out carefully at different time points and centrifuged for 3 min at 1000 rpm. The cells were washed two times in RPMI medium and lysed in sterile water. The lysates were plated on 7H10 agar plates and kept at 37 °C for 4 days.

### 2.8. Biofilm Estimation

*M. smegmatis* strains (Ms_tig, Ms_VC and Ms_WT) were grown in 7H9 media supplemented with 0.05% Tween 80, 10% OADC and 0.001% glycerol. The cultures were diluted to an OD_600_ of 0.08 and incubated at 37 °C in a test tube. Further, 20 ng/mL of anhydrotetracycline was added to all of the *M. smegmatis* strains. To allow pellicle development at the liquid-air interface, *M. smegmatis* strains were subsequently incubated as static culture for 7 days in growth medium without Tween 80.

Biofilm formation was quantified using the crystal violet assay [22]. Anhydrotetracycline-induced Ms_tig, Ms_VC and Ms_WT were cultured as static phase in a sterile, flat-bottomed 96-well microtiter plate. After 7 days, the media below the pellicle was aspirated out. Further, 125 μL of 0.1% crystal violet dye was used to stain the pellicle. The stained pellicle was then washed with water three times before adding 30% acetic acid. The plates were incubated for 15 min at room temperature, before recording the absorbance spectrophotometrically at 550 nm.

### 2.9. Prediction of Antigenicity

In silico analysis of the antigenicity of *M.tb*-*tig* was performed with a tool developed by the Immunomedicine group of UCM, Spain. This tool utilizes the method described to determine the antigenic peptides [23]. The occurrence of amino acid residues in previously established segmental epitopes has been tabulated and forms the basis of the prediction. A minimum of eight residues is essential for a segment to be considered significant.

### 2.10. Immune Assay

The presence of immunogenic proteins induces the secretion of cytokines from macrophages. Cytokine secretion levels were assayed by Enzyme-Linked Immunosorbent Assay (ELISA) method as previously reported [21,24]. THP-1 cells were differentiated into macrophages by adding PMA (20 ng/mL) for 24 h in 5% CO_2_ at 37 °C [20,25,26]. Then, differentiated THP-1 cells (2 × 10^4^ cells/cm^2^) were treated with *M.tb*-tig protein (2.5–20 µg) for 48 h. LPS was used as positive control while heat-denatured *M.tb*-tig was used as negative control. The culture supernatants were used to quantify the levels of cytokines using ELISA as described earlier [17].

## 3. Results

### 3.1. rTig of M.tb Is Enzymatically Active

**Figure 1:**
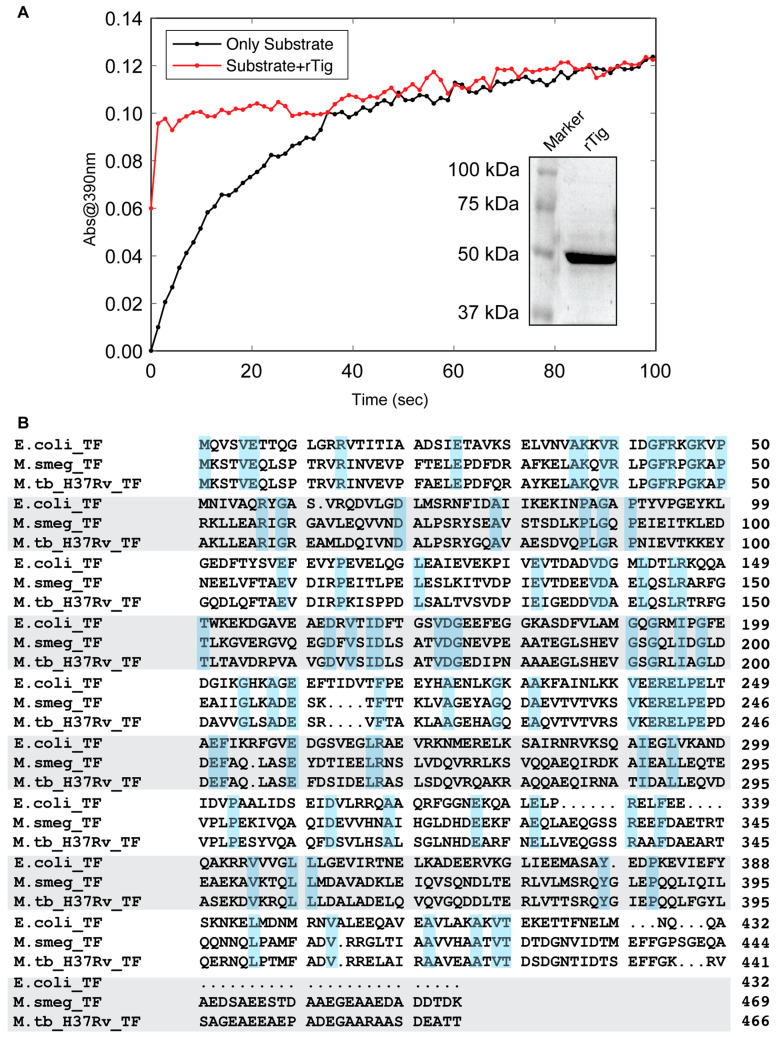
Purified rTig is enzymatically active. (**A**): Isomerization activity of rTig was measured using a small chromogenic peptide and α chymotrypsin as coupled enzyme and juxtaposed with the rate of spontaneous cis-trans isomerization in the absence of the rTig. Purified rTig was found to weigh around 50 kDa on 10% SDS PAGE. (**B**): Alignment of trigger factors of Escherichia coli K-12, *M. smegmatis* and *M. smegmatis* H37Rv using T-Coffee protein alignment server of EMBL-EBI. The matching residues are highlighted.

The molecular mass of purified recombinant *M.tb* rTig was found to be 50 kDa on 10% SDS-PAGE (Figure 1A). α chymotrypsin and a chromogenic peptide were used in a coupled assay to assess the enzyme activity of rTig. The rise in the isomerization activity rate before reaching saturation shows rTig is active (Figure 1A). Alignment of amino acid sequence of trigger factors of *E. coli* K-12, *M. smegmatis* and *M.tb* H37Rv shows the similarities among the three. As usual, *M. smegmatis* trigger factor has higher match with *M.tb* than *E. coli* K-12 (Figure 1B).

### 3.2. rTig Displays Properties of a Chaperone Protein

**Figure 2:**
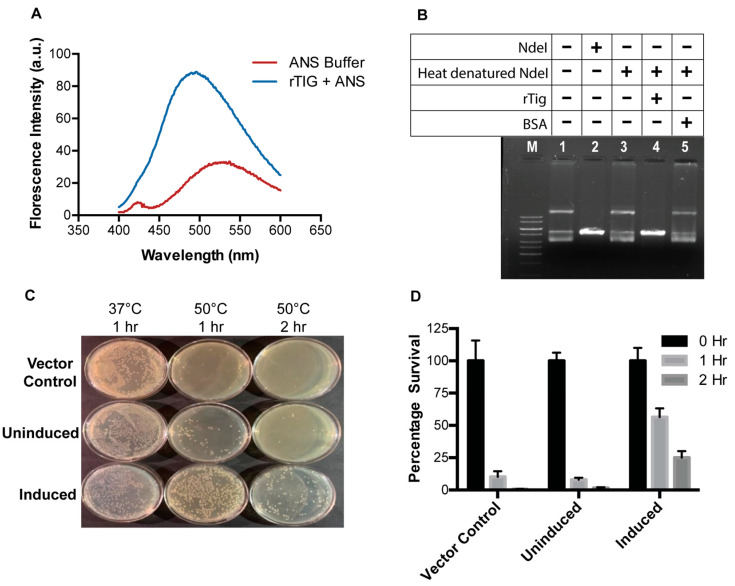
*M.tb Rv2462c* (rTig) exhibits in vitro and in vivo chaperone-like activity. (**A**): ANS-bound to *M.tb Rv2462c* (rTig) confirmed surface hydrophobicity. Fluorescence excitation was monitored in the range of 400–650 nm. Fluorescence spectra of control (ANS buffer) and ANS bound to rTig were recorded in red and blue colour, respectively. (**B**): rTig protected heat-denatured NdeI protein M, GeneRuler 1KB marker. Lane 1, multiple bands of undigested pET22b (5.3 kb), Lane 2, digested pET22b by NdeI, Lane 3, digested pET22b by heat denatured NdeI. Lane 4, digested by thermally denatured NdeI in the presence of *Rv2462c* protein. Lane 5, digested pET22b by heat-denatured NdeI in the presence of BSA protein. (**C**,**D**): rTig rescues *E. coli* cells from heat shock: Transformed *E. coli* cells (with pET22b alone and pET22b-*Rv2462c*) underwent heat treatment (50 °C). At one and two hours, bacterial growth was determined by the CFU count. *E. coli* expressing *Rv2462c* displayed a greater number of CFU compared to vector control.

Previous studies have established the importance of surface hydrophobicity for chaperone activity [17]. 1-anilinonaphthalene-8-sulfonate (ANS) is a hydrophobic selective dye bond with cationic groups of polyamino acids and proteins by the formation of ion pairs. It binds to the hydrophobic sites buried in a protein surface and has long been used for characterizing protein-binding sites. The sulfonate group of ANS interacts with cationic groups of lysine and arginine, causing a hypsochromic shift, thereby, enhancing fluorescence intensity and blue shift of fluorescence emission [27]. In our study, a shift in the emission wavelength, associated with enhanced fluorescence intensity was observed in case of ANS bound to rTig (Figure 2A). This observation establishes the presence of surface hydrophobicity in rTig, which is an attribute of chaperone-like proteins.

Protecting proteins from thermal aggregation and preserving their functional activity is characteristic to most chaperones [17]. The chaperone function of rTig was investigated by examining its ability to restore the enzymatic activity of the heat denatured NdeI. After heat denaturation, NdeI failed to digest pET22b plasmid (Figure 2, lane 3), but retained the activity after addition of rTig (lanes 4). BSA, used as negative control, failed to display a similar restorative effect on heat-denatured NdeI (Figure 2B; lane 5). Restoration of enzymatic activity of heat-denatured NdeI by rTig suggests its role in protein refolding.

To understand the chaperone-like role of *M.tb*-rTig, in vitro, we examined if the *M.tb*-rTig has the ability to rescue *E. coli* BL21 (DE3) strain after exposure to heat stress. There was no significant difference in the CFU count between uninduced or vector control strains (Figure 2C,D). We observed a significant increase in the CFU count of the induced culture as compared to the uninduced and vector control after an hour of heat shock (Figure 2D). The number of CFU decreased when incubated for two hours, but was still significantly higher than controls. These data suggest that rTig can protect the bacterium from thermal stress and might function as chaperone.

### 3.3. M.tb Tig Helps Mycobacterial Survival within THP-1 Macrophages

**Figure 3:**
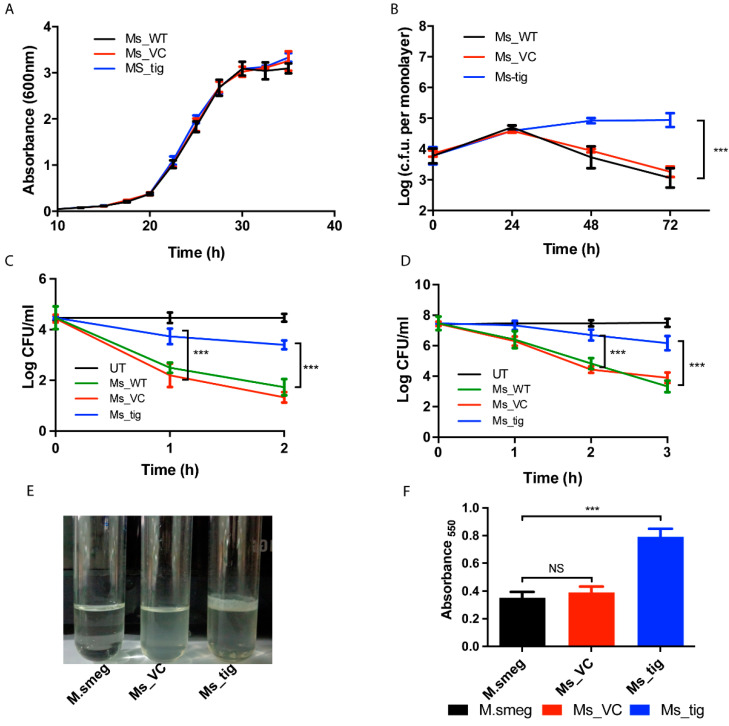
Role of rTig in stress adaptation and biofilm production. (**A**): *M. smegmatis* carrying rTig shows no change in growth characteristics, in vitro. Bacterial growth (OD_600_) of Ms_WT, Ms_VC and Ms_tig was plotted. Data are the mean ± standard deviation of data derived from three distinct experiments. (**B**): *M. smegmatis* expressing *M.tb Rv2462c* show enhanced survival within THP-1 cells. Human THP-1 cells were infected with *M. smegmatis* mc^2^ 155 (Ms_WT), *M. smegmatis* vector control (Ms_VC) and those transformed with *M.tb Rv2462c* (Ms_tig) and plated at different time intervals. The log of c.f.u per monolayer of THP-1 cells was computed at designated time points. The results represent three individual experiments (*** *p* < 0.001). They show the mean ± SEM of three triplicate wells. (**C**,**D**): Growth characteristics of *M. smegmatis* under temperature and oxidative stress. Ms_WT, Ms_VC and Ms_tig cultures were grown up to an O.D. of 0.4. They were then subjected to different temperature (50 °C or 37 °C) (panel-C) H_2_O_2_ treatment (panel-D) and log c.f.u. was computed after pre-determined time intervals. The results shown represent three individual experiments (*** *p* < 0.001) and are the mean ± SEM of three triplicate wells. (**E**,**F**): rTig encoded by *M.tb Rv2462c* promotes the development of pellicle and production of biofilm in *M. smegmatis*. Upon induction with anhydrotetracycline to express rTig of *M.tb*, pellicle formation at the liquid air junction was observed and quantified in M.smeg, Ms_VC and Ms_tig.

We aim to determine the influence of *M.tb*-*Rv2462c* on the ability of mycobacterium to adapt and survive in host cells. Under normal conditions, the overexpression of *M.tb*-Tig (Ms_Tig) has no impact on the growth pattern of *M. smegmatis* compared to controls (Ms_VC, Ms_WT) (Figure 3A).

To assess the role of *M.tb*-Tig on the survival of the pathogen inside host cells, we compared CFU of *M. smegmatis* strains (Ms_Tig, Ms_VC and Ms_WT) after infecting THP-1 cells. A steady rise in the CFU count of Ms-Tig was observed up to 72 h (*p* < 0.001) while a decline in the CFU was observed for Ms_WT and Ms_VC after 24 h (Figure 3B). Our observations suggest that rTig contributes to intracellular adaptation and growth of mycobacterium in human macrophages.

Intracellular mycobacteria usually experience oxidative stress. For examining the possible impact of *M.tb*-Tig on the survival of *M. smegmatis* under hydrogen peroxide stress, we treated the *M. smegmatis* cultures with 7 mM of H_2_O_2_ for 3 h. The higher CFU count in Ms_Tig compared to Ms_VC and Ms_WT strains suggest that *M.tb*-Tig offer protection against oxidative stress. (Figure 3D) and might play a role in stress adaptation of the mycobacterium. For heat stress experiment, different *M. smegmatis* strains grown till 0.6 OD600 with and without anhydrotetracycline were exposed to 50 °C temperature for 2 h. Further, survival under temperature stress is a significant attribute of chaperone proteins. *M. smegmatis* expressing *M.tb* trigger factor show improved survival under a temperature stress of 50 °C compared to controls (Figure 3C).

To further consolidate the contribution of *M.tb* tig in virulence and stress adaptation of the mycobacteria, we studied the possible role of the protein in modulating biofilm formation. As described previously, *M. smegmatis* expressing rTig, wild-type *M. smegmatis* and Ms_VC, used as control, were induced when the cells were grown both with and without anhydrotetracycline. The results demonstrated a noteworthy contrast in biofilm production by the three strains. Enhanced biofilm formation of nearly 2-fold (*p* < 0.005) was exhibited by Ms_Tig (Figure 3E,F). This suggests that *M.tb*. rTig is involved in the development of biofilms.

### 3.4. Exposure of THP-1 Cell with M.tb Tig Elicited Pro-Inflammatory Responses

**Figure 4:**
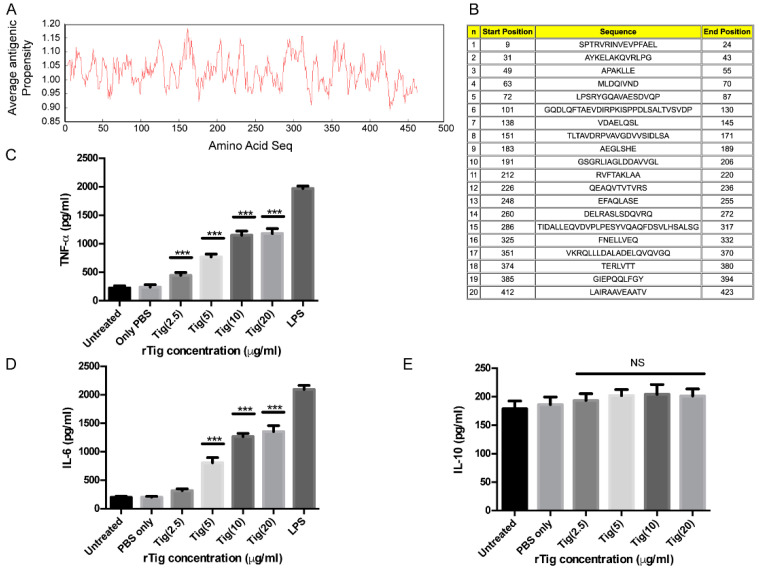
(**A**): Analysis of rTig using antigenicity prediction tool. (**B**): Major antigenic stretches. (**C**), (**D**,**E**): *M.tb Rv2462c* enhances the production of pro-inflammatory cytokines by human monocytes. Concentration-dependent rise in the secretion of pro-inflammatory cytokines ((**C**): TNF-α; (**D**): IL-6) as a function of treatment of THP-1 cells with rTig. No significant changes were observed in IL-10 secretion. The mean ± SD of three replicates has been represented here. LPS was taken as the positive control. (*** *p* < 0.001).

Antigenicity profiling using the antigenicity prediction tool revealed the amino acid sequence of *M.tb* Tig exhibits high antigenicity and multiple antigenic stretches (Figure 4A,B). Furthermore, we examined the role of *M.tb*-rTig in the regulation of different cytokine secretions. LPS, the endotoxin contaminant of the recombinant protein rTig was removed by polymyxin B agarose beads. The differentiated THP-1 cells were treated with different dosage (2.5 to 20 µg/mL) of *M.tb*-Tig. The dose-dependent increased levels of TNF-α and IL-6 were observed by the treatment of *M.tb*-Tig. These results revealed that *M.tb*-*Rv2462c* elicited an increased secretion of proinflammatory cytokine by macrophages (Figure 4C,D). However, no significant changes were observed in the levels of IL-10 (Figure 4E). These results are comparable to the immune-modulatory function of other mycobacterial PPIases [20].

## 4. Discussion

The current study was aimed at functionally characterizing the *M.tb* trigger factor rTig and exploring its role in *M.tb* pathogenesis by studying its contribution to immunomodulation and stress adaptation of the pathogen. The disease progression and outcome of tuberculosis are influenced by a complicated interplay between the virulence factors of the pathogen and immune response of the host. Alongside their role in protein folding and secretion, cell signalling and apoptosis, bacterial chaperones are involved in virulence and stress adaptation. Additionally, in this study, we have tried to focus precisely on these moonlighting activities of the trigger factor protein of *M.tb*. Bacterial pathogens such as *M.tb*, *H. pylori* and *Clostridium difficile*, use chaperones such as Hsp60 and Hsp70 as adhesins, to attach to the host tissue. Regulation of host cell activity by cell signalling has been exhibited by molecular chaperones such as Cpn60 and Hsp70. Cpn60, Hsp70, Hsp90 and Cpn10 display extracellular signalling in pathogens such as *M.tb*, *M. paratuberculosis*, *E. coli*, *Chlamydia trachomatis* and *H. pylori*. It has been demonstrated earlier that *M.tb* secretes Cpn10 when inside the phagolysosome of the macrophage. The overexpression of Hsps in a mutant of the intracellular pathogen, *S. typhimurium* has previously illustrated the importance of chaperones in stress adaptation [28,29,30,31].

The trigger factor, encoded by the *tig* gene, is highly conserved across diverse bacterial species. This ribosome-associated molecular chaperone was initially discovered in vitro, in Gram-negative bacteria, folding the precursor of the outer membrane protein A (pro-OmpA) into a conformation suitable for translocation [19,32]. It has now been linked to the virulence of many pathogens. In this study, we investigated the chaperone-like activity and immunological role of the trigger factor protein of *M.tb*. The binding of molecular chaperones to misfolded proteins is guided by surface hydrophobicity. The distinctive blue shift in the emission maxima, and enhanced absorbance by ANS-binding to rTig suggests the presence of surface hydrophobicity similar to chaperones. Earlier studies have reported that the trigger factor protects against thermal denaturation in *E. coli* [33]. Our current study demonstrates that *M.tb tig* protects the cells against high-temperatures and aids in the restoration of enzyme activity of thermally denatured proteins. Incubation with rTig restores enzyme activity of NdeI, pointing towards its expected role in protein refolding. After getting satisfactory evidence of rTig’s chaperone-like nature, in vitro, we proceeded to investigate the activity of the protein, in vivo. Additionally, indeed, *E. coli* transformed with rTig displays a significantly increased survival after heat shock as compared to the controls.

To assess the role in stress adaptation and to demonstrate chaperone-like activity, the heterologous expression of *M.tb* trigger factor in *E. coli* and *M. smegmatis* expression systems was carried out. Using the vector alone and uninduced controls, as well as the fact that they do have sequence and therefore structural variations, the influence of the trigger factors of the expression systems was accounted for.

*M.tb* has the ability to resist stress and survive inside the host. We explored the contribution of rTig in protecting *M. smegmatis* cells under conditions of oxidative stress. In comparison to *M. smegmatis* controls, we observed a significant increase in the survival of *M. smegmatis* strain expressing *M.tb* tig. Our observations lie in line with in vitro results, indicative of rTig acting as a chaperone-like protein which also contributes to stress adaptation.

The overproduction of pro-inflammatory cytokines by human macrophages is considered to be a hallmark of tuberculosis. Antimicrobial activity against the pathogen is affected primarily by granulomas, whose formation and maintenance are believed to be modulated by TNF-α. Previous studies have also linked TNF-α production with intracellular viability and virulence [34]. IL-6 is involved in inflammation and pathology and is thus believed to play a role in disease progression in tuberculosis [35,36]. THP-1 cells were treated with increased doses of rTig, which amplified the secretion of pro-inflammatory cytokines such as TNF-α and IL-6. However, no significant changes were observed in the secretion of anti-inflammatory cytokine, IL-10. *M. smegmatis* cells transformed with rTig displayed enhanced survival in THP-1 cells. This points towards a crucial role of rTig in the pathobiology of tuberculosis. There are seemingly paradoxical role of pro-inflammatory cytokines vis a vis host benefits/survival. Pro-inflammatory cytokines could be pro-host as having anti-bacterial effects and anti-host as being toxic to host. Additionally, in case of tuberculosis, pro-inflammatory cytokines released earlier in the infection result in accumulation of immune cells around the granuloma resulting in spread of the pathogen.

IL-6 is not a pure pro-inflammatory, but rather has an anti-inflammatory tinge as it inhibits endotoxin-induced TNF-α and IL-1 production in mononuclear cells and induce the soluble receptors for TNF-α which is its natural inhibitors [37]. Furthermore, the macrophage polarization is no longer considered to be existing in the dichotomy of pro and anti-inflammation. They exist in the more flexible and dynamic in response to the macroenvironment and may exist in the intermediate polarization state rather than canonical binaries of pro- and anti-states [38].

The *E. coli* trigger factor also enhances the binding efficiency of the chaperone GroEL [19,39,40]. In Gram-positive bacteria *Streptococcus pyogenes*, the homologue of the trigger factor, RopA, is critical for the secretion and maturation of SpeB [41,42]. Deficiency of LuxS, a modulator of biofilm formation and acid and oxidative stress tolerance in *S. mutans,* was found to induce the upregulation of its trigger factor, strongly suggesting its involvement in the virulence of the pathogen [19,43]. In the swine pathogen *Streptococcus suis serotype* 2, *tig* encodes a virulence regulator, associated with the expression of the pathogen’s virulence genes. The *tig* mutants of *S. suis serotype 2* influence its adaptive capacity against environmental stresses [19].

Previous research demonstrated the role of *Mycobacterium abscessus* and *P. aeruginosa* in the production of biofilms in cystic fibrosis as well as a virulence factor in UPEC isolates. The likelihood of biofilm development within the host tissues is suggested by the occurrence of extracellular *M.tb* in biofilm-such as structures inside the lung lesions of guinea pigs infected with *M.tb* [44]. In the current study, *M. smegmatis* expressing rTig significantly upregulates biofilm production, in comparison to the strains lacking rTig. Our findings establish the involvement of rTig in biofilm production.

Cell surface proteins play crucial role in the regulation of biofilm formation. Tig (*Rv2462c*) has been reported in the cell wall fraction along with cytosolic fraction in different proteomic profiling [45,46,47]. The Tig overexpression in *M. smegmatis*, then, might have altered its physiochemical properties or working as a surface-associated adhesin-like molecule, promoting biofilm formation. For example, localization of Lap A protein to cell surface enhances the biofilm formation Pseudomonas fluorescens [48]. Similarly, BpfA–BpfG–BpfD proteins governs biofilm formation in *Shewanella oneidensis* [49]. Some previous studies in *Staphylococcus aureus* have shown role of Tig biofilm formation. Tig acts as a chaperone protein and help in folding of proteins essential in biofilm formation [50].

The results of this study on *M.tb tig* is in accordance with the findings of previous studies. Surface hydrophobicity and the ability to restore enzyme activity of heat-denatured NdeI establishes the expected chaperonic nature of rTig. The rTig protein considerably increases survivability under high-temperature conditions, which was suggestive of its involvement in the virulence of the pathogen. This was further established by the observations that the presence of the protein distinctly increased tolerance to oxidative stress (H_2_O_2_).

## 5. Conclusions

Our study demonstrates the chaperonic nature and immunomodulatory potential of the *M.tb* trigger factor protein. Our findings also establish its contribution to tolerance and adaptation to host-generated stresses, implying its involvement in the infection cycle of *M.tb*. Revelations of these attributes of *M.tb*-Tig highlights its significance in *M.tb* virulence and make it an important target for novel therapeutic interventions against tuberculosis, thus fulfilling the primary aim of this study. Further studies would provide a better comprehension of the mechanisms employed by the protein to enable the intracellular lifestyle and enhanced pathogenicity of *M.tb*.

## Data Availability

Not applicable.
